# Syringaldehyde Exhibits Antibacterial and Antioxidant Activities against *Mycobacterium marinum* Infection

**DOI:** 10.3390/microorganisms12020348

**Published:** 2024-02-07

**Authors:** Da Wen, Chaoqun Meng, Yazhi Feng, Lin Shen, Yiyao Liu, Wei Sun, Guangxin Chen, Changxin Wu

**Affiliations:** 1 Institutes of Biomedical Sciences, Shanxi University, Taiyuan 030006, China; wdbio@outlook.com (D.W.); kellyliu0914@163.com (Y.L.); chengx@sxu.edu.cn (G.C.); 2 Shanxi Provincial Key Laboratory of Medical Molecular Cell Biology, Shanxi University, Taiyuan 030006, China; 3 Shanxi Provincial Key Laboratory for Prevention and Treatment of Major Infectious Diseases, Shanxi University, Taiyuan 030006, China

**Keywords:** syringaldehyde, antibacterial, oxidative stress, *Mycobacterium marinum*, zebrafish, NRF2

## Abstract

Tuberculosis (TB) is caused by infection with *Mycobacterium tuberculosis* (*Mtb*), which has a unique resistance to many antimicrobial agents. TB has emerged as a significant worldwide health issue because of the rise of multidrug-resistant strains causing drug-resistant TB (DR-TB). As a result, the development of new drugs or effective strategies is crucial for patients with TB. *Mycobacterium marinum* (*Mm*) and *Mtb* are both species of mycobacteria. In zebrafish, *Mm* proliferates and forms chronic granulomatous infections, which are similar to *Mtb* infections in lung tissue. Syringaldehyde (SA) is a member of the phenolic aldehyde family found in various plants. Here, we investigated its antioxidative and antibacterial properties in *Mm*-infected cells and zebrafish. Our results demonstrated that SA inhibits *Mm*-infected pulmonary epithelial cells and inhibits the proliferation of *Mm* in *Mm*-infected zebrafish, suggesting that SA provides an antibacterial effect during *Mm* infection. Further study demonstrated that supplementation with SA inhibits the production of malondialdehyde (MDA) and reactive oxygen species (ROS) and increases the levels of reduced glutathione (GSH) in *Mm*-infection-induced macrophages. SA inhibits the levels of MDA in *Mm*-infected zebrafish, suggesting that SA exerts antioxidative effects in vivo. Additionally, we found that SA promotes the expression of NRF2/HO-1/NQO-1 and the activation of the AMPK-α1/AKT/GSK-3β signaling pathway. In summary, our data demonstrated that SA exerts antioxidative and antibacterial effects during *Mm* infection both in vivo and in vitro and that the antioxidative effects of SA may be due to the regulation of NRF2/HO-1/NQO-1 and the AMPK-α1/AKT/GSK-3β signaling pathway.

## 1. Introduction

Tuberculosis (TB) is a respiratory disease caused by infection with *Mycobacterium tuberculosis* (*Mtb*), and it has claimed more human lives throughout history than any other microorganism-induced disease [1]. According to the 2023 WHO TB report, an estimated 10.6 million people were infected with *Mtb*, resulting in 1.6 million global deaths in 2022. The TB incidence rate saw a 3.6% increase from 2020 to 2021, marking a reversal of the decades-long decline of about 2% [2]. Therefore, TB continues to pose a significant global public health threat to humans [3]. Moreover, TB caused by infection with drug-resistant and multidrug-resistant strains has exacerbated the difficulties in treatment [4]. Consequently, exploring novel drugs against TB may provide a viable approach to mitigate this disease. *Mycobacterium marinum* (*Mm*) is a pathogen that is a close genetic relative to *Mtb* [5]. *Mm* is a major cause of infection in freshwater and marine fish and can also cause infection in humans [6]. Most of the patients with *Mm* infection have a history of contact with seafood, and their occupations are mainly fishers, seafood sellers, and fish fanciers, because of the higher possibility of contact with a contaminated aqueous environment or with fish [7,8]. *Mm* causes necrotizing granuloma-like TB in fish and granulomatous lesions in human skin and deep tissue [9]. The duration of therapy for *Mm* infection is three to six months for immunocompetent patients; immunocompromised patients and immunosuppressed patients require more time [10]. Antituberculosis drugs are used clinically to treat *Mm* infection, and previous studies have shown that an anti-TB compound was discovered by *Mm* high-throughput screening [11]. Zebrafish are seen as a great animal model for exploring mycobacterial pathogenesis [12,13]. Zebrafish have been used as a rapid method for screening anti-TB drugs [14,15]. Here, we use zebrafish to screen an anti-TB drug and investigate its antibacterial effect on *Mm*.

It has long been postulated that macrophages are the primary phagocytic cells for *Mtb* [16,17]. Although macrophages exhibit a potent antibacterial function, *Mtb* replicates within the cytoplasm subsequent to its escape from the phagosome [18]. Upon recognition and phagocytosis of *Mtb* by phagocytes, the activation of the niacinamide adenine phosphate oxidase 2 (NOX2) receptors on the phagocyte membrane is triggered, subsequently inducing the production of reactive oxygen species (ROS) [19]. The appropriate concentration of ROS enhances protection against bacterial infection [20], and it can directly kill pathogens by causing oxidative damage to biological components such as DNA and proteins or indirectly kill pathogens by activating downstream non-oxidative mechanisms as a signaling molecule [21]. However, a persistent *Mtb* infection can result in an elevation of ROS levels, leading to oxidative stress and inflammatory response, subsequently impairing tissue and organ function and exacerbating TB progression [22]. Previous studies have demonstrated that *Mtb* infection induced high levels of oxidative stress in vitro and in vivo [23,24]. Therefore, targeting oxidative stress may represent a promising avenue for alleviating TB.

**Figure 1:**
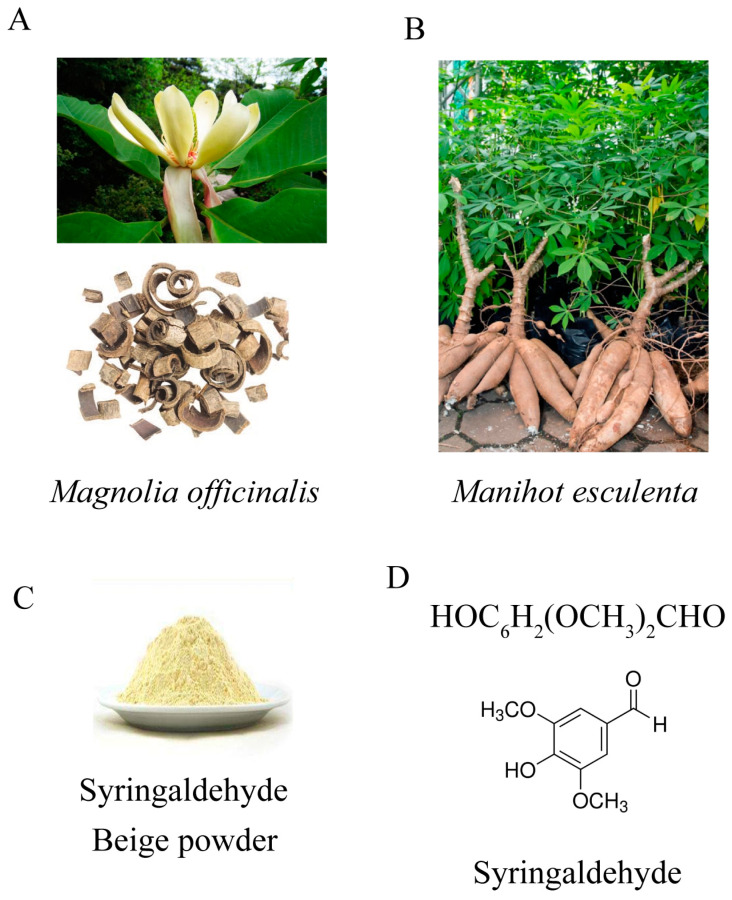
(**A**) *Magnolia officinalis*. [online image], 2018, 360doc. www.360doc.com/content/18/0429/09/42668302_749620463.shtml, accessed on 29 April 2018. (**B**) *Manihot esculenta*. [online image], 2018. http://www.360doc.com/content/22/0830/10/153132_1045851273.shtml, accessed on 7 December 2018. (**C**) SA, a light beige powder. [online image]. www.chemicalbook.com/SupplyInfo_1024189.htm, accessed on 16 March 2021. (**D**) The chemical structure of SA (3,5-dimethoxy-4-hydroxybenzaldehyde, C_9_H_10_O_4_).

Syringaldehyde (SA, 3,5-dimethoxy-4-hydroxybenzaldehyde), a member of the phenolic aldehyde family, is a compound that presents naturally in minute quantities but has been widely found in various plants such as *Manihot esculenta* and *Magnolia officinalis* [25] (Figure 1). As a traditional Chinese medicine, it exhibits diverse biological properties. In vitro studies have demonstrated that SA inhibits the growth of *Staphylococcus aureus*, *Klebsiella pneumonia*, and *Pseudomonas aeruginosa* [26]. SA affects the type III secretion system of *Salmonella enterica serovar* Typhimurium and impedes bacterial invasion into Hela cells [27]. Moreover, SA demonstrates antioxidative and antiapoptotic effects in rats with cerebral ischemia injury [28], while also exhibiting the inhibition of inflammation, oxidation, and histopathological alterations in rats with cardiotoxicity [29]. SA also exhibits an anti-hyperglycemic effect in streptozotocin-induced diabetic rats [30]. Additionally, it exerts antiproliferative effects on colon cancer cells [31] and possesses antitumorigenic properties [32]. In summary, SA possesses antimicrobial, antioxidative, anti-hyperglycemic, anticancer, and anti-inflammatory properties. However, the effects of SA on *Mm* remain unexplored. Therefore, the purpose of this paper is to explore the impact of SA on *Mm* infection and its associated impact on oxidative stress.

## 2. Materials and Methods

### 2.1. Syringaldehyde

Syringaldehyde (Sigma-Aldrich, S7602, St. Louis, MO, USA), a light beige powder, 98% purity, was dissolved in dimethyl sulfoxide (DMSO) to a concentration of 0.5 M. DMSO was used as a reference in subsequent experiments.

### 2.2. Mycobacterium marinum and Cell Culture

*Mycobacterium marinum* M strain (ATCC BAA-535) was kindly provided by Professor Chen Niu (Fudan University), and tdTomato *Mm* originated from our laboratory [33]. *Mm* were cultured in Middlebrook 7H9 (BD, New York, NY, USA) supplemented with 10% oleic acid albumin dextrose catalase (OADC) (BD) at a temperature of 30 °C.

The A549 and RAW264.7 cell lines were obtained from the BeNa Culture Collection company (Beijing, China). A549 and RAW264.7 cells were cultured in RPMI1640 and Dulbecco’s modified Eagle medium (DMEM) medium (Gibco, Grand Island, NY, USA) supplemented with 10% fetal bovine serum (FBS) (Sorfa, Beijing, China), respectively. All cells were cultured at 37 °C with 5% CO_2_ in a humidified chamber.

Isolation of primary peritoneal macrophages: A total of 4 mL of 3% thioglycollate broth was injected into the peritoneal cavity of WT and NRF2^−/−^ mice. Three days later, all mice were sacrificed. Subsequently, 4 mL of DMEM medium containing 10% heat-inactivated FBS was injected into the peritoneal cavity, and the abdomen was gently massaged for 5 min. The resulting peritoneal lavage fluid was collected and centrifuged (1000 rpm, 5 min). The supernatant was discarded, and the cells were resuspended with DMEM medium containing 10% FBS and cultured at 37 °C with 5% CO_2_. Two hours later, non-adherent cells were removed by replacing them with fresh complete medium, and the remaining adherent cells were primary peritoneal macrophages.

### 2.3. Cell Viability Assay

The A549 and RAW264.7 cells were cultured in a complete medium including different concentrations of SA (0.1, 0.2, 0.3, 0.4, 0.5, 1, and 2 mM) for 24 h. Subsequently, the cells were treated with 10 μL cell counting kit 8 (cck8) solution (Yeasen, Shanghai, China) for 30 min. Following this incubation period, the absorbance at an optical density of 450 nm was measured using a microplate reader.

### 2.4. Cells and Zebrafish Infection

A549 and RAW264.7 cells were cultured until they reached approximately 80% confluency, followed by treatment with 0.5 mM SA or an equal volume of DMSO for 1 h. Subsequently, the cells were supplemented with *Mm* for a duration of 4 h before being removed and washed with cold phosphate-buffered solution (PBS) containing gentamycin to eliminate any bacteria present in the medium. The infected cells were fixed with 4% paraformaldehyde (PFA) and then incubated with DAPI for nuclear staining. The intracellular *Mm* was observed using an LSM710 confocal microscope (Carl Zeiss, Jena, Germany).

Zebrafish, AB strain, were imported from the China Zebrafish Resource Center. A total of 60 zebrafish larvae at the well-developed stage (72 h) were injected with *Mm* at a concentration of 100 CFUs/nL via caudal vein microinjection. Subsequently, the infected zebrafish were cultured for seven days in media containing 0.5 mM SA or an equal volume of DMSO (30 fish, respectively), after which the bacterial load within the fish was observed using a confocal microscope.

### 2.5. Colony-Forming Unit (CFU) Assay

After infection, the cells were detached from the dish using a cell scraper and centrifuged to remove the supernatant. Then, the cells were treated with PBS containing 0.01% Triton X-100 (Solarbio, Beijing, China) and vigorously agitated to lyse the cells and release *Mm*. The cell lysate was diluted and plated onto Middlebrook 7H10 (BD) agar plates and incubated at 30 °C for approximately 14 days. Infected zebrafish were immersed in PBS containing 3% kanamycin (Solarbio) and incubated for 45 min at a temperature of 27 °C. Thereafter, a cell tissue crusher was employed to lyse the infected zebrafish and release *Mm*. The zebrafish lysate was subsequently coated onto 7H10 agar plates and incubated for 2 weeks at a temperature of 30 °C.

### 2.6. RNA Isolation and Quantitative Reverse Transcription PCR (qRT-PCR)

Total RNA was extracted from cells using RNAiso plus (Takara, Tokyo, Japan) following the manufacturer’s protocol. Subsequently, 1 μg of total RNA was used to generate cDNA using PrimeScript RT Master Mix (Takara). qRT-PCR was performed using a LightCycler 480 Instrument (Roche, Basel, Switzerland) using Real-time PCR super mix SYBR green (Mei5 Biotechnology, Beijing, China). The β-actin, IL-6, IL-1β, and TNF-α primer sequences were as previously described [34]. The IL-17a primer sequences were as follows: F: 5′-TTTAACTCCCTTGGCGCAAAA-3′; R: 5′-CTTTCCCTCCGCATTGACAC-3′.

### 2.7. Western Blotting Assay

The detailed experimental procedures can be found in our previous study [35]. Antibodies used included p-AKT, AKT, NRF2, and iNOS from Cell Signaling Technology (Danvers, MA, USA) and NQO-1, HO-1, COX-2, p-AMPK-α1, AMPK-α1, p-GSK-3β, GSK-3β, β-actin, and goat anti-rabbit or goat anti-mouse horseradish peroxidase (HRP)-conjugated secondary antibody from ABclonal (Wuhan, China).

### 2.8. Reactive Oxygen Species (ROS) Assay

After 4 h of infection with *Mm*, the culture medium was removed, and the cells were washed with PBS to eliminate extracellular *Mm*. Then, serum-free medium was added, and the cells were cultured for 24 h. Subsequently, the medium was replaced with serum-free medium containing DCFH-DA (10 μM/L) and incubated for 20 min. Subsequently, the cells were washed with serum-free medium to remove any remaining DCFH-DA in the medium. The levels of ROS within the cells were measured using flow cytometry (CytoFLEX S, Beckman, Brea, CA, USA) and a fluorescence microscope (Nikon ECLIPSE Ti2-U). The detailed experimental procedures were conducted according to the instructions provided by ROS Assay Kit (Solarbio, Beijing, China).

### 2.9. Malondialdehyde (MDA) Assay

The lysis of 5 million cells or 0.1 g tissue was performed using a cell tissue crusher (JXFSTPRP-24, Shanghai jing xin, Shanghai, China) with 1 mL of extracting solution. The cracking procedure involved applying a power of 200 W for 3 s with an interval of 10 s, repeated for a total of 30 cycles. After centrifugation at 8000× *g* and 4 °C for 10 min, the supernatant was collected. Subsequently, the levels of MDA in cells and tissue were measured using an assay kit (Solarbio, Beijing, China), following the provided instructions.

### 2.10. Reduced Glutathione (GSH) Assay

Ice bath ultrasound lysis was performed by using 1 mL of extracting solution to lyse 5 million cells. The cracking procedure involved applying a power of 200 W for 3 s with an interval of 10 s, repeated for a total of 30 cycles. The supernatant was collected by centrifugation at 8000× *g* and 4 °C for 10 min. Subsequently, the levels of GSH in cells and tissues were measured by using the GSH assay kit (Solarbio, Beijing, China), following the manufacturer’s instructions.

### 2.11. Statistical Analysis

The data are presented as mean ± SEM. One-way analysis of variance (ANOVA) was used to analyze significant effects, followed by Tukey’s HSD test, and the *t*-test was performed for two groups (* *p* < 0.05, ** *p* < 0.01, *** *p* < 0.001). All experiments were carried out with three independent replications.

## 3. Results

### 3.1. SA Suppresses Mycobacterium marinum (Mm) Invasion into Lung Epithelial Cells

**Figure 2:**
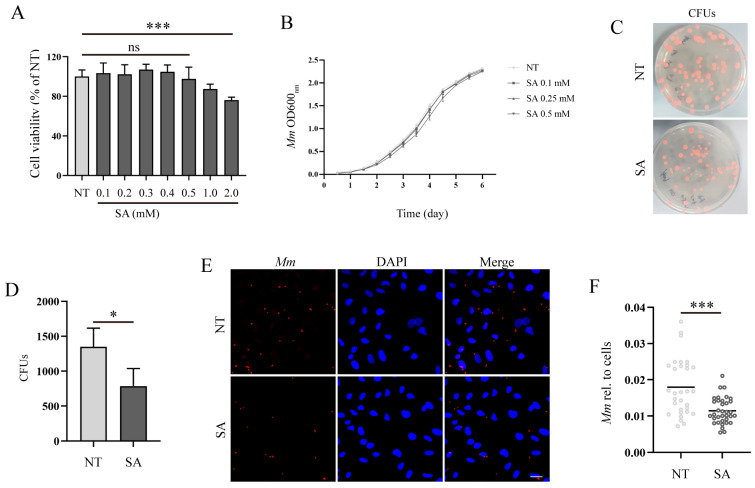
SA increases the resistance of lung epithelial cells to *Mm* infection. (**A**) The effect of various concentrations of SA on A549 cell viability; “ns” denotes “no significance” (*n* = 6). (**B**) The growth curve of *Mm* cultured with 0.1, 0.25, and 0.5 mM SA for 6 days (*n* = 3). (**C**,**D**) The CFU assays analyzing the effects of SA on *Mm* infection in A549 cells (*n* ≥ 9). (**E**) The efficiency of *Mm* infection in cells with pretreatment of 0.5 mM SA was determined by confocal microscope. *Mm* (red) represents tdTomato *Mm*, DAPI (blue) represents cell nuclei. Multiplicity of infection (MOI) = 10:1; the images are 20×; scale bar: 20 μm (*n* ≥ 31). (**F**) The *Mm* immunofluorescence intensity was quantified using ImageJ software; the results represent the area of *Mm*/the area of the cell nucleus (*n* ≥ 31). Means ± SEM; * *p* < 0.05 and *** *p* < 0.001.

To investigate the antimicrobial effects of SA, we initially evaluated the impact of SA on the viability of A549 cells. The results obtained from the CCK8 assays revealed that a concentration of 0.5 mM SA almost did not influence the cell viability of A549 cells, whereas concentrations of 1 and 2 mM of SA noticeably reduced cell viability (Figure 2A). Consequently, a concentration of 0.5 mM SA was selected for subsequent experiments. Prolonged treatment with SA for 6 days did not exert any significant influence on the *Mm* growth curve (Figure 2B). Subsequently, we assessed the impact of SA on *Mm* infection in human lung epithelial cells (A549). A CFU assay was employed to analyze the effects of SA after *Mm* infection, the outcomes revealed that A549 cells with SA pretreatment had much less intracellular *Mm* (Figure 2C,D). The cells with *Mm* infection were determined by confocal microscopy, and the images were statistically analyzed with Image J 1.44p software; the results demonstrated that the amount of intracellular *Mm* in A549 cells with SA pretreatment was significantly less than that in cells without SA treatment (Figure 2E,F). The in vitro experiments described above indicate that while SA does not directly affect *Mm* growth, it effectively impedes *Mm* invasion into lung epithelial cells or increases host cell resistance to *Mm* infection.

### 3.2. SA Inhibits the Inflammatory Response Induced by Mycobacterium marinum (Mm) Infection

**Figure 3:**
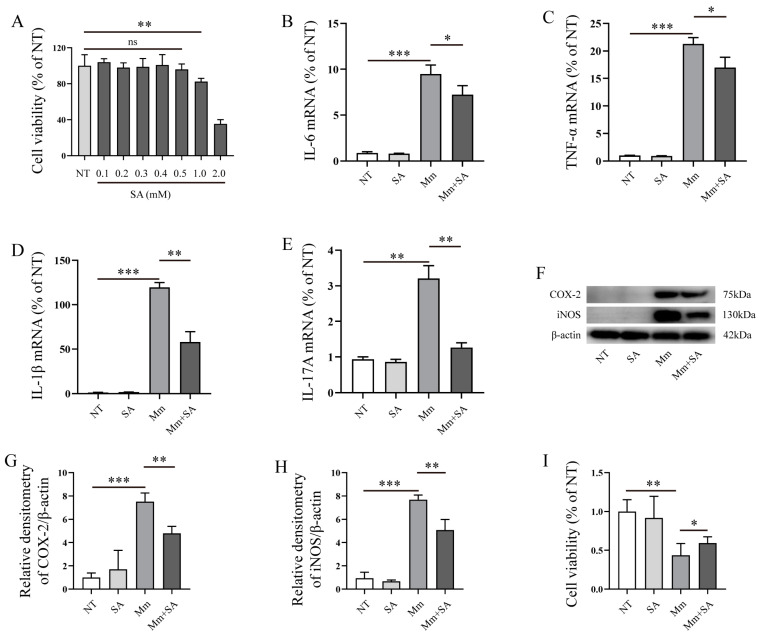
SA inhibits the inflammatory response induced by *Mm* infection. (**A**) The effect of different concentrations of SA on RAW264.7 cell viability; “no significance, ns” (*n* = 6). (**B**–**E**) qRT-PCR detection of the mRNA levels of IL-6, TNF-α, IL-1β, and IL-17A in RAW264.7 cells after *Mm* infection or the addition of 0.5 mM SA (*n* = 3). (**F**–**H**) Western blotting analysis of the effects of 0.5 mM SA on iNOS and COX-2 expression in macrophages after *Mm* infection (*n* = 3). (**I**) RAW264.7 cell viability, cells treated with *Mm* and 0.5 mM SA-pretreated *Mm*. Means ± SEM; * *p* < 0.05, ** *p* < 0.01, and *** *p* < 0.001.

Next, we explored the effects of SA on the inflammatory response in RAW264.7 cells after *Mm* infection. Prior to commencing the experiment, we initially evaluated the impact of SA on RAW264.7 cell viability. Cell viability assays revealed that 0.5 mM SA had no negative effects on the RAW264.7 cell viability, whereas concentrations of 1 and 2 mM noticeably decreased cell viability (Figure 3A). Therefore, 0.5 mM of SA was selected as the working concentration. Our results demonstrated that SA significantly decreased the expression of IL-6, TNF-α, IL-1β, and IL-17A in *Mm*-infected RAW264.7 cells (Figure 3B–E). Western blotting assays demonstrated a marked reduction in the expression of iNOS and COX-2 in macrophages which were supplemented with SA after *Mm* infection (Figure 3F–H). The results of the cell viability assays showed that the pretreatment of *Mm* with SA improved the *Mm*-infected cell viability in comparison with cells infected with untreated *Mm* (Figure 3I).

### 3.3. SA Alleviates Oxidative Stress in Mycobacterium marinum (Mm)-Infected Macrophages

**Figure 4:**
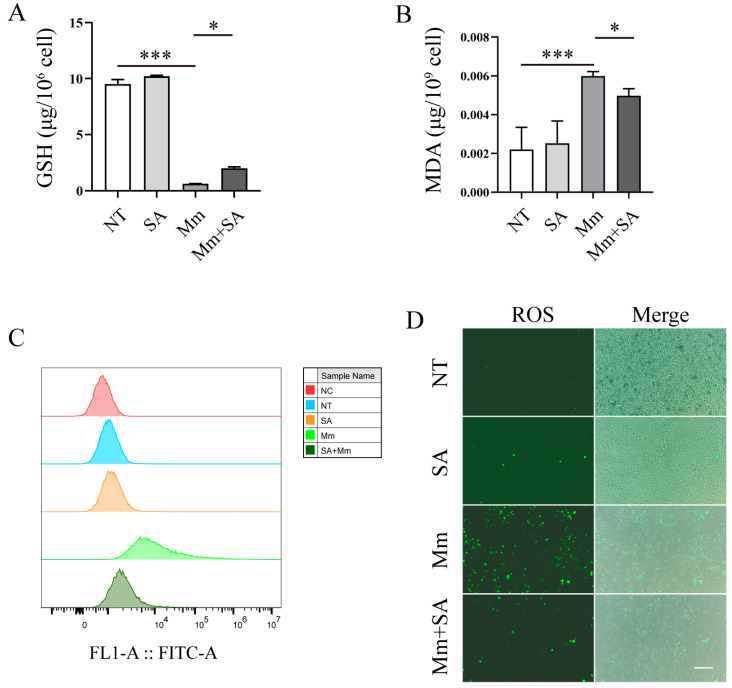
SA alleviates oxidative stress in *Mm*-infected macrophages. (**A**,**B**) The cellular levels of MDA and GSH in SA-treated and *Mm*-treated macrophages (*n* = 3). (**C**,**D**) The effects of SA on the production of ROS in *Mm*-infection-induced macrophages, determined using flow cytometry and fluorescence microscopy (*n* = 3); the images are 20×; scale bar: 50 μm. Means ± SEM; * *p* < 0.05 and *** *p* < 0.001.

Subsequently, we explored the effects of SA on *Mm*-infection-induced oxidative stress. Our experiments demonstrated that *Mm* infection decreased the content of GSH in macrophages, whereas supplementation with 0.5 mM SA significantly alleviated the decrease (Figure 4A). And 0.5 mM SA significantly decreased the levels of MDA in *Mm*-infected macrophages (Figure 4B). Additionally, while *Mm* infection promoted the production of ROS, supplementation with 0.5 mM SA markedly decreased the production of ROS in *Mm*-infected macrophages (Figure 4C,D). All the results above suggest that SA possesses a potent antioxidant property in macrophages after *Mm* infection.

### 3.4. SA Inhibits Mycobacterium marinum (Mm) Proliferation and Alleviates Mm-Infection-Induced Oxidative Stress in Zebrafish

**Figure 5:**
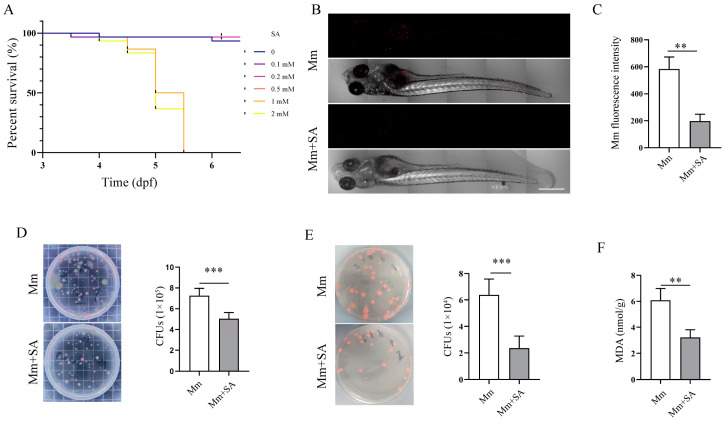
SA inhibits *Mm* proliferation and alleviates *Mm*-infection-induced oxidative stress in zebrafish. (**A**) The effects of different doses of SA on the survival of zebrafish larvae. (**B**) Imaging of *Mm*-infected zebrafish using laser scanning confocal microscope; the images are 10×; scale bar: 500 μm. (**C**) The statistics of B results. (*n* ≥ 6) (**D**) CFU assays analyzing the impact of 0.5 mM SA on the *Mm* load in zebrafish (*n* = 6). (**E**) CFU assays analyzing the effect of 0.5 mM SA on A549 cells after *Mm* infection. (**F**) The levels of MDA in the tissue homogenate of SA-treated and *Mm*-infected zebrafish (*n* = 3). Means ± SEM; ** *p* < 0.01 and *** *p* < 0.001.

The infection of zebrafish with *Mm* is a natural TB model for the investigation of TB pathogenesis and screening anti-TB drugs, and this model was used to further validate the antioxidant and antibacterial effects of SA on *Mm* infection. Before commencing the experiment, we initially assessed the impact of SA on the survival, development, and growth of zebrafish larvae. First, we tested the working concentration of SA for its treatment of zebrafish larvae; it was found that a concentration of 0.5 mM SA had no discernible effects on the development and growth of zebrafish (Figure 5A). Subsequently, a concentration of 0.5 mM SA was used as the working concentration and added to the E3 medium of zebrafish larvae followed by infection with *Mm* via caudal vein microinjection. The infection process lasted 7 days; thereafter, we directly observed and quantified the *Mm* in zebrafish. The images revealed that 0.5 mM SA effectively reduced *Mm* load in zebrafish (Figure 5B,C). To further determine whether the fluorescent intensity correlates with bacilli load inside zebrafish, CFU assays were employed, and it was verified that the supplementation with SA decreased the *Mm* load in zebrafish (Figure 5D). These findings indicated that SA restricts the proliferation of *Mm* in zebrafish. Simultaneously, the addition of SA was observed to inhibit *Mm* proliferation in A549 cells after *Mm* infection (Figure 5E). In vivo, the assays for the quantification of MDA demonstrated that SA reduced the levels of MDA in zebrafish (Figure 5F). The experiments described above demonstrate that SA protects against *Mm* infection and subsequently results in less oxidative stress in zebrafish.

### 3.5. SA Activates AMPK-α1/AKT/GSK-3β Signaling Pathway and Promotes NRF2/HO-1/NQO-1 Protein Expression

**Figure 6:**
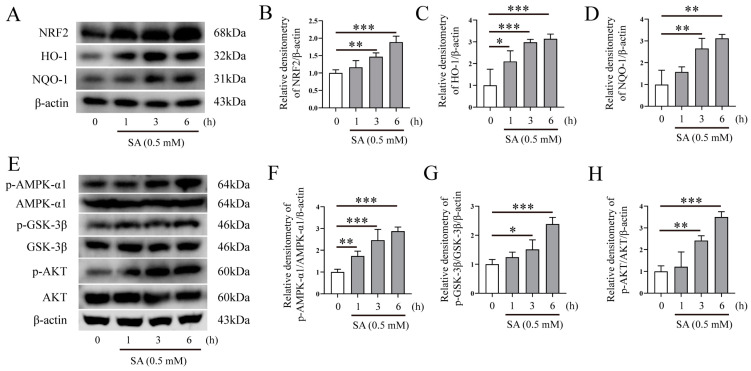
SA activates AMPK-α1/AKT/GSK-3β and NRF2/HO-1/NQO-1 signaling pathways. (**A**–**D**) Western blotting analysis of the effects of 0.5 mM SA on NRF2, HO-1, and NQO-1 protein expression in RAW264.7 cells (*n* = 3). (**E**–**H**) Western blotting analysis of the effects of 0.5 mM SA on the AMPK-α1, GSK-3β, and AKT signaling pathway in RAW264.7 cells (*n* = 3). Means ± SEM; * *p* < 0.05, ** *p* < 0.01, and *** *p* < 0.001.

To investigate the mechanism of SA underlying its activity against oxidative stress in *Mm*-infected cells, we examined the effect of SA on NRF2 and the AMPK-α1/AKT/GSK-3β signaling pathway. Western blotting assays revealed that the 0.5 mM SA treatment led to a time-dependent upregulation of NRF2, HO-1, and NQO-1 protein expression (Figure 6A–D), suggesting that SA may exert an antioxidative effect by activating the NRF2 signaling pathway after *Mm* infection. Our results also demonstrated that SA treatment improved the phosphorylation of AMPK-α1, AKT, and GSK-3β (Figure 6E–H). Collectively, our results indicate that SA exerts antioxidative effects during *Mm* infection by improving the phosphorylation levels of the AMPK-α1, AKT, and GSK-3β signaling pathway and upregulating the expression of the NRF2, HO-1, and NQO-1 proteins.

### 3.6. The Antioxidative Property of SA Depends on NRF2 in Mycobacterium marinum (Mm)-Infected Macrophages

**Figure 7:**
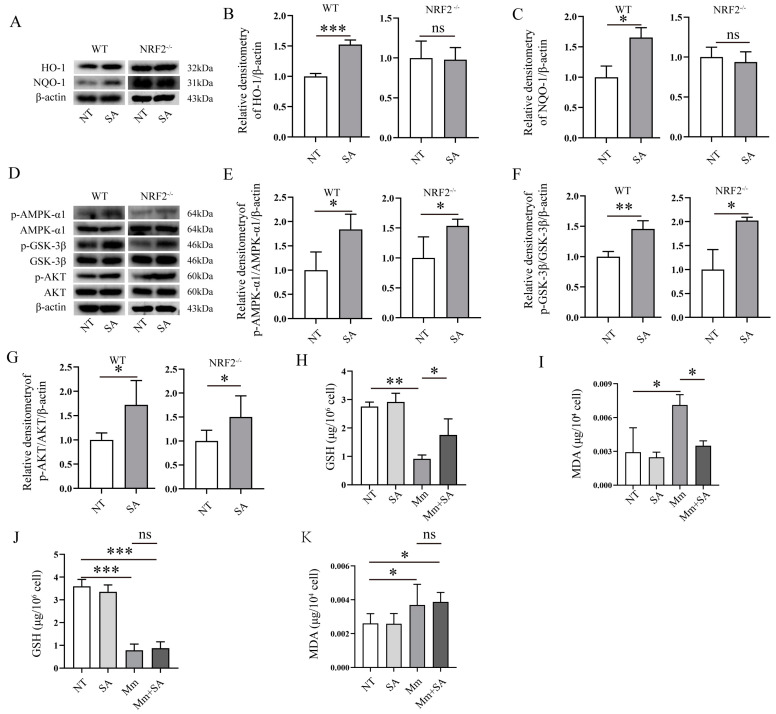
The antioxidative activity of SA depends on NRF2 in *Mm*-infected macrophages. (**A**–**C**) Western blotting analysis of the effects of 0.5 mM SA on the protein expression of HO-1 and NQO-1 in *Mm*-infected macrophages isolated from WT and NRF2^−/−^ mice (*n* = 3). (**D**–**G**) Western blotting analysis of the effects of 0.5 mM SA on the AMPK-α1, GSK-3β, and AKT signaling pathway in *Mm*-infected macrophages isolated from WT and NRF2^−/−^ mice (*n* = 3). (**H**,**I**) The levels of GSH and MDA in *Mm*-infected macrophages isolated from WT mice (*n* = 3). (**J**,**K**) The levels of GSH and MDA in *Mm*-infected macrophages isolated from NRF2^−/−^ mice (*n* = 3). Means ± SEM; no significance, ns; * *p* < 0.05, ** *p* < 0.01, and *** *p* < 0.001.

We postulated that the level of NRF2 expression may affect or correlate with oxidative stress in cells after bacterial infection. To investigate the role of NRF2 in the antioxidant effect of SA during *Mm* infection in macrophages, we tested if NRF2 knockout affects the antioxidative properties of SA in macrophages after mycobacterium infection. Primary peritoneal macrophages were isolated from both wild-type (WT) and NRF2^−/−^ mice. Next, we measured the effects of SA on the NRF2-related signaling pathways. The results showed that the addition of SA enhanced the expression of HO-1 and NQO-1 proteins in the macrophages isolated from WT mice but had no effects in cells from NRF2^−/−^ mice (Figure 7A–C), whereas the supplementation with SA significantly activated the AMPK-α1, GSK-3β, and AKT signaling pathway in macrophages isolated from both WT and NRF2^−/−^ mice (Figure 7D–G). We also tested the GSH and MDA levels induced by *Mm* infection in the macrophages isolated from WT and NRF2^−/−^ mice, and the results showed that supplementation with SA had no effects on the levels of GSH and MDA in *Mm*-infected macrophages isolated from NRF2^−/−^ mice (Figure 7J,K) but had an effect on the levels of GSH and MDA in cells from WT mice (Figure 7H,I). Overall, our results suggest that NRF2 acts as a critical regulator for the antioxidative role of SA during *Mm* infection.

## 4. Discussion

The zoonotic disease tuberculosis (TB) is caused by *Mtb* infection and affects both humans and livestock. It primarily spreads through the respiratory tract via the inhalation of air containing TB bacilli from infected individuals [36,37]. Despite a gradual decline in TB incidence over the years, it remains the leading cause of mortality worldwide among infectious diseases [38]. Unquestionably, TB continues to be a significant global public health issue for humans [3,39]. Moreover, the ability of *Mtb* strains to develop drug resistance has led to the emergence of drug-resistant tuberculosis (DR-TB), multidrug-resistant tuberculosis (MDR-TB), and extensively drug-resistant tuberculosis (XDR-TB) strains [40], thereby presenting healthcare providers with increasingly challenging obstacles in achieving effective treatment. This not only signifies the risk of mortality but also hampers achieving “The global plan to end TB”. Therefore, the search for novel and effective anti-TB drugs remains an urgent imperative. *Mm* is also a pathogen that can affect both humans and animals, and it is very closely related to *Mtb* based on 98% genome sequence homology [10,41]. *Mm* has functional elements similar in organization to of those in *Mtb* that trigger a host’s oxidative stress response [42]. And *Mm* can survive within macrophages by preventing phagosome maturation [43]. *Mm* proliferates in zebrafish and forms chronic granulomatous infections, which closely resemble *Mtb* infections in lung tissue [44], and zebrafish infected with *Mm* serve as a natural model for investigating TB pathogenesis and screening potential anti-TB drugs [12,14,15]. In this study, our objective was to investigate the antimicrobial and antioxidative effects of SA on *Mm*-infected cells and zebrafish and preliminarily elucidate the mechanism underlying SA’s property of activity against oxidative stress in these infection models.

SA inhibits the growth of *Staphylococcus aureus*, *Klebsiella pneumonia*, and *Pseudomonas aeruginosa* [26] and impedes *Salmonella enterica serovar* Typhimurium invasion into Hela cells [27]. In our study, we found that supplementation with SA has no effects on the growth of *Mm*, while noticeably inhibiting *Mm* invasion into the lung epithelial cells. This lack of effect on *Mm* growth may be attributed to the arabinogalactan-containing cell wall of *Mm*, which serves as a natural barrier between *Mycobacterium* and antibiotics [45]. Next, a series of experiments in our study demonstrated that SA suppresses the production of proinflammatory cytokines and inhibits the expression of iNOS and COX-2 in macrophages after *Mm* infection, parallel to the protective role of SA in isoproterenol-induced cardiotoxicity rats [29], suggesting it has potential anti-inflammatory properties. It was reported that SA improved the activity of superoxide dismutase (SOD) and the levels of nuclear respiratory factor-1 (NRF1) and inhibited the levels of MDA in rats with cerebral ischemia injury [28]. Consistent with the previous studies [28,29], our results showed that SA reduces the production of ROS and the levels of MDA while improving the levels of GSH in *Mm*-infected macrophages. To further validate the antimicrobial and antioxidative properties of SA, we assessed its effects on bacilli load and oxidative stress in zebrafish. Fluorescent scanning and CFU results showed that supplementation with SA restricts the proliferation of *Mm* in zebrafish. We observed a decrease in MDA levels upon supplementation with SA in *Mm*-infected zebrafish. Above all, our study showed that SA exerts antibacterial and antioxidative effects on *Mm* infection either in vitro or in vivo.

NRF2 plays a crucial role in inflammation, autophagy, oxidative stress [46], drug detoxification, metabolic reprogramming, protein stasis, and unfolded protein response [47] and is associated with a variety of diseases, including TB [48] and cancer [49]. The objective of this study was to investigate the role of NRF2 in SA’s suppression of oxidative stress induced by *Mm* infection. The GSK-3β signaling pathway has been identified as a novel regulator of NRF2, and GSK-3β and PI3K/AKT can be activated by AMPK signaling [50], indicating that the AMPK-α1/AKT/GSK-3β signaling pathway could potentially mediate NRF2’s antioxidative property [50], subsequently inhibiting mitochondrial oxidative stress [51]. In order to investigate the mechanism underlying SA’s attenuation of oxidative stress in macrophages after *Mm* infection, we evaluated the impact of SA on the abovementioned signaling pathways after *Mm* infection. Our results revealed that SA improves NRF2, HO-1, and NQO-1 protein expression; improves the activation of the AMPK-α1, AKT, and GSK-3β signaling pathway; reduces the production of MDA; and ameliorates the reduction of GSH in macrophages isolated from WT mice. However, the supplementation with SA did not elicit any effects on HO-1 and NQO-1 protein expression and had no effect on the levels of MDA and GSH; conversely, it significantly improved the phosphorylation of AMPK-α1, GSK-3β, and AKT in macrophages isolated from NRF2^−/−^ mice, suggesting that SA effectively exerts its antioxidative effect during *Mm* infection by targeting the AMPK-α1, AKT, GSK-3β, and NRF2 signaling pathway. Experiments conducted with NRF2^−/−^ mice demonstrated that SA mitigates oxidative stress via its regulation of NRF2 expression. Further elucidation of the molecular mechanism underlying SA’s upregulation of NRF2 expression which ameliorates oxidative stress after mycobacterial infection will produce more interesting information for us to better understand the pathogenesis of *Mm* infection and develop a more effective strategy for countering pathogenic mycobacteria.

## 5. Conclusions

The treatment of *Mm* infection remains uncertain because of the different host infection cases and the natural multidrug-resistance properties of *Mm*. Proper oxidative stress is beneficial for clearing bacteria in mycobacterial infections, whereas excessive oxidative stress is harmful to the host. Our findings demonstrated that SA inhibits the invasion of *Mm* into lung epithelial cells and reduces oxidative stress in macrophages, and its antimicrobial and antioxidative properties were also observed in *Mm*-infected zebrafish. Additionally, our results demonstrated that SA exerts an antioxidative effect on *Mm* infection by activating the AMPK-α1/AKT/GSK-3β signaling pathway and upregulating the expression of NRF2/HO-1/NQO-1. Importantly, compared with conventional anti-TB drugs, SA offers the benefit of being readily available and less prone to causing bacterial drug resistance. Our results reveal the antioxidative and antibacterial effects of SA on *Mm*-infected cells and zebrafish, providing a novel strategy for the treatment of *Mm* infections.

## Data Availability

Data are contained within the article.

## Abbreviations

ANOVA	analysis of variance
CCK8	cell counting kit 8
CFU	colony-forming unit
DMEM	Dulbecco’s modified Eagle medium
DMSO	dimethyl sulfoxide
DR-TB	drug-resistant TB
FBS	fetal bovine serum
GSH	glutathione
HRP	horseradish peroxidase
MDA	malondialdehyde
*Mm*	*Mycobacterium marinum*
MOI	multiplicity of infection
NOX2	niacinamide adenine phosphate oxidase 2
MDR-TB	multidrug-resistant tuberculosis
*Mtb*	*Mycobacterium tuberculosis*
NT	non-treated
OADC	oleic acid albumin dextrose catalase
PBS	phosphate-buffered solution
PFA	paraformaldehyde
qRT-PCR	quantitative reverse transcription PCR
ROS	reactive oxygen species
SA	syringaldehyde
SEM	standard error of the mean
TB	tuberculosis
WT	wild type
XDR-TB	extensively drug-resistant tuberculosis
